# Waste not, want not: upcycling research data from chronic pain trials

**DOI:** 10.1097/PR9.0000000000001070

**Published:** 2023-04-04

**Authors:** Anna Woodbury

**Affiliations:** a Division of Pain Management, Department of Anesthesiology, Emory University School of Medicine Atlanta, GA, United States,; b Center for Visual and Neurocognitive Rehabilitation (CVNR), Atlanta VA Healthcare System, Atlanta, GA, United States

## Abstract

Jacobsen SM, Moore T, Douglas A, Lester D, Johnson AL, Vassar M. Discontinuation and nonpublication analysis of chronic pain randomized controlled trials. PAIN Rep 2023;8:e1069.

## Abstract 2

The article by Jacobsen et al. highlights an alarming trend of unpublished trials in chronic pain. This commentary theorizes ways to reduce waste and upcycle data.

## 1. The problem of research waste

The article by Jacobsen et al. brings attention to chronic pain research and rates of nonpublication, trial discontinuation, and research waste. The authors show that 40% of clinical trials in chronic pain go unpublished (higher rates of non-publication for industry-sponsored studies) and make a strong case for encouraging publication, point out factors that contribute to completion and publication, and suggest that we should reduce research waste by making data available. While I agree with the sentiments shared, there are significant barriers to solving this problem of research waste. Regardless, the first step towards solving a problem is to acknowledge that there is one, which the article unarguably does.

Before commencing a clinical trial, investigators in the United States must register their trials in ClinicalTrials.gov. ClinicalTrials.gov requires that investigators provide updates to the registered trial and report the results of their trial, once available. However, other than a series of somewhat annoying reminder emails from the ClinicalTrials.gov team, there are few repercussions of ignoring these requirements. The authors analyzed more than 400 randomized controlled trials registered in ClinicalTrials.gov and performed their best to reach out to investigators regarding reasons for nonpublication and trial discontinuation. They noted that industry-sponsored studies and medical device studies had higher rates of discontinuation and nonpublication, possibly because participants experienced adverse events and the results would be unfavorable to the sponsor's business model.

How do we address this problem? In the current environment where funding is proportionally on par if not increasing from industry relative to federal and nonprofit organizations,^2^ it would be hard to discourage industry-sponsored trials at the risk of discouraging clinical research altogether. Should we provide incentives for publication or dis-incentives for nonpublication? Should we increase taxes to support government sponsorship of clinical trials? It is probably beyond my abilities to completely solve this issue, but I can certainly underscore the important questions raised by this article.

## 2. Ideals vs reality

Where Jacobsen et al. are somewhat short-sighted is in their idealist approach to research. As an example, they note that 76% of randomized controlled trials (RCTs) are discontinued because of poor recruitment^1^ and insinuate that this is not a justifiable reason because it is preventable. While I completely agree that RCTs should ideally not be discontinued or left unfinished and that stopping rules should be put in place before the trial begins rather than as an arbitrary decision later in the course of the study, the idea that poor recruitment is entirely preventable is overly optimistic. Poor recruitment is a sign that the study is no longer feasible, and despite the most thoroughly anticipated potential pitfalls and alternative strategies, sometimes unexpected events (eg, a COVID-19 pandemic) hit, and recruitment halts. This is especially true for rarer diseases or prolonged studies that require a high participant burden. Government agencies, oversight committees, and funders are likely to stop a trial rather than try to recover sunk costs. Continuing such a trial would be more wasteful for the continued financial costs as well as the increasing strain on the trialists to recruit for a study that is beyond their scope to complete.

The authors note some potential associations with trial completion and publication, which include motivated investigators, quality leadership, and well-trained staff. Unfortunately, the assessment of investigators and well-trained staff is somewhat subjective, and while past work might predict future success, this is not always the case. Furthermore, it is difficult for authors to publish negative results because many journals tend not to publish these types of studies. Fortunately, in recent years, there have been an increasing number of publishers willing to fill this gap, with journals specifically targeting negative results.

While I believe that there are significant barriers to trial completion and successful publication, I agree 100% with the authors that whatever data have been collected should still be shared, regardless of whether the trial is complete or how the results may affect the sponsor. Sharing reasons for trial failure or discontinuation can help future investigators decide whether they would pursue a similar investigation, encounter the same problems, and have a plan to overcome such issues. Sharing of the data could allow a separate group of investigators to pool results from a similar data collection for the purposes of a meta-analysis. Ultimately, data sharing can ensure that any information collected is appropriately up-cycled to reduce research waste and lead to more informed decision-making.

## 3. Upcycling chronic pain research data

I learned about upcycling from a children's show (My Little Pony), but I like the term in the context of reducing research waste. Upcycling means to recycle or reuse something in a way that increases its value and makes it better than the original. An example of upcycling is mentioned in the article: the ENIGMA (Enhancing Neuroimaging Genetics through Meta-Analysis) Consortium. The ENIGMA Consortium has recently established a Chronic Pain Working Group, with the aim of collecting data sets from researchers across the globe who sign a memorandum of understanding that allows them to share and analyze collected data sets to create meta-analyses. This is especially necessary in neuroimaging data because image acquisitions can be both time-consuming and costly, and data sharing allows neuroimagers to increase the sample size and power of their findings. Incomplete data sets and raw data are welcome. Proper credit is attributed to the working group members, allowing cross-collaborative efforts and increased publication rates. However, such a method of upcycling data still depends on motivated investigators who are willing to share and participate in such a working group.

One possible solution to encourage data sharing is to make it part of administrative and regulatory oversight. For example, local institutional review boards (IRBs) could require a data sharing plan as part of the protocol, with a stipulation that data be shared even if the trial is terminated. Many federally funded studies already have a data sharing plan requirement as part of their research proposals. However, to enforce a data sharing plan for industry-sponsored studies, it may be necessary to make it part of the IRB review because all clinical trials must pass through an IRB, regardless of funding source. An alternative is to provide a monetary incentive for data sharing, or, conversely, a penalty that may be incurred through ClinicalTrials.gov. These methods depend more on regulatory agencies and government bodies rather than on individual investigators, and it is not clear to me to what degree they would be enforceable.

## 4. Conclusions and further questions

The study by Jacobsen et al. raises many important points in the discussion of chronic pain research and research waste. While Jacobsen et al. scoured ClinicalTrials.gov and therefore assessed studies registered in the United States, it is important to acknowledge that there are at least 24 clinical trial registries across the globe that meet World Health Organization Trial Registration criteria.^3^ It would be interesting to investigate whether the trend noted in the United States is present in other countries, as well, and whether there are rules, regulations, or cultural expectations that lead to a relative increase or decrease in publication rates. There are unanswered questions that need to be further addressed, and we need awareness from our community that leads to action to ensure that data are not being purposely hidden or neglectfully discarded. “Insanity is repeating the same mistakes and expecting different results,” per the classic quote misattributed to Albert Einstein (but likely from a Narcotics Anonymous pamphlet circa 1981). It is important to know why clinical trials fail, what results were collected before discontinuation, and whether there were any adverse events, to prevent the same mistakes from being made again. Without knowing what mistakes have already been made, we will be practicing insanity rather than science.

## Disclosures

The author has no conflict of interest to declare.
